# Pulmonary benign metastasizing leiomyoma from uterine leiomyoma

**DOI:** 10.1186/1477-7819-11-163

**Published:** 2013-07-18

**Authors:** Sufeng Chen, Yawei Zhang, Jie Zhang, Hong Hu, Yufan Cheng, Jianhua Zhou, Lei Shen, Haiquan Chen

**Affiliations:** 1 Department of Thoracic Surgery, Fudan University Shanghai Cancer Center, 270 Dong’an Road, Shanghai 200032, China; 2 Department of Pathology, Fudan University Shanghai Cancer Center, 270 Dong’an Road, Shanghai 200032, China; 3 Department of Oncology, Shanghai Medical College, Fudan University, 270 Dong’an Road, Shanghai 200032, China

**Keywords:** Benign neoplasms, Castration, Metastasis, Pulmonary, Rare diseases

## Abstract

**Background:**

Benign metastasizing leiomyoma (BML) occurs in a low proportion of uterine leiomyomas and treatment methods for BML are diverse and controversial. The study introduces preliminary experiences in the diagnosis and treatment of BML with the purpose of finding a suitable management strategy for these patients.

**Methods:**

Three patients with BML were treated in our department from April 2008 to July 2012. Each of these patients presented with multiple nodules in both lungs, where we performed video-assisted thoracoscopic wedge resection to harvest enough tissue for histopathologic and immunohistochemical examination. The patients were treated with medical castration or surgical castration after the diagnosis of BML.

**Results:**

The ultimate pathologic results ruled out the possibility of leiomyosarcoma and other metastatic diseases, and confirmed that the pulmonary lesions were BML. The lung lesions remained stable in two patients who were treated by surgical castration, and the lung nodules regressed in one patient treated with gonadotropin-releasing hormone analogues.

**Conclusions:**

The diagnosis of BML is based on the medical history of uterine myomas and histopathologic and immunohistochemical examination of lung nodules. Video-assisted thoracoscopic wedge resection is the best way to harvest tissue for diagnosis. The better outcomes in BML seem to call for medical intervention, either chemical or surgical, after diagnosis is made.

## Background

Metastasis is the capacity of cancer cells to spread from a primary site to form tumors at distant sites in the body; it is the hallmark of malignant disease. However, some non-cancerous tumors, despite their benign appearance, show a low-grade clinical malignant behavior and present a rare manifestation. Uterine leiomyoma, the most common form of uterine tumors, is considered a benign tumor. However, it may present with an unusual growth pattern termed benign metastasizing leiomyoma (BML), which occurs in a low proportion of uterine leiomyomas. Due to the low morbidity and scarcity of reports on this condition, there is no consensus on which methods should be used to treat this disease. Herein, we present three cases of Chinese women with extensive lung metastases after uterine leiomyoma resection, and introduce our preliminary experiences in the diagnosis and treatment of BML. These patients might benefit from castration, either chemical or surgical, after diagnosis of BML. Written informed consent was obtained from the patient for the publication of this report and any accompanying images.

## Methods

Three patients with benign metastasizing leiomyoma have been treated in our department from April 2008 to July 2012. They all underwent a pulmonary wedge resection by video-assisted thoracoscopic surgery (VATS), histopathological and immunohistochemical examinations were carried out in all patients. The patients were treated with medical castration or surgical castration after the diagnosis of BML. The detailed information are given below.

### Case 1

**Figure 1:**
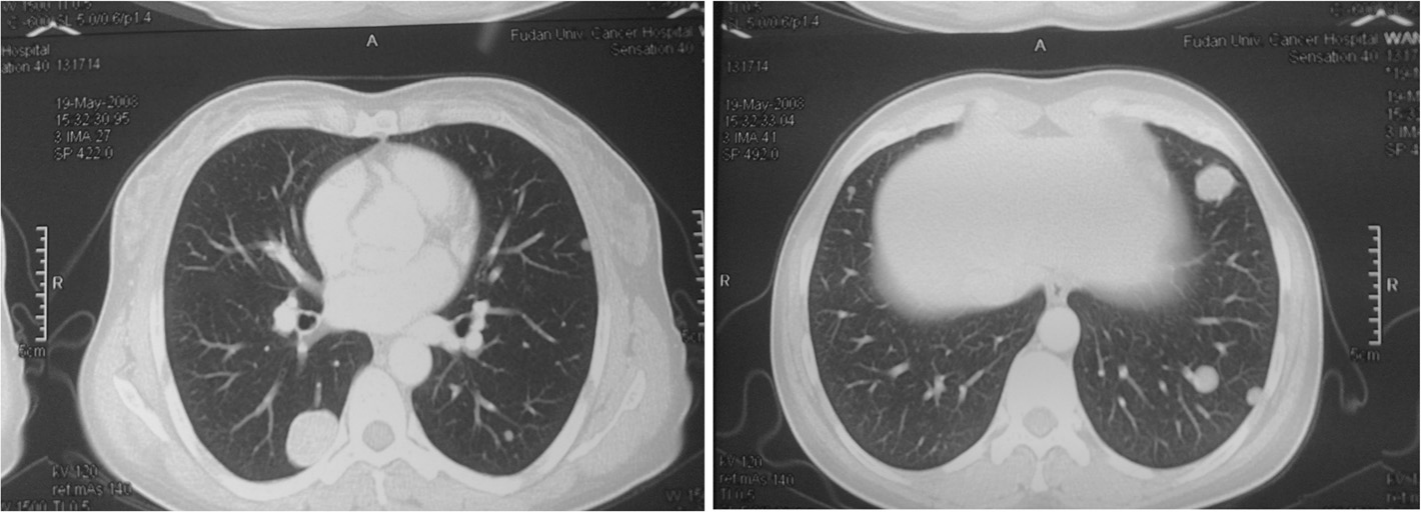
Computed tomography scan shows that lesions are generally smooth and diffusely distributed in both lungs.

**Figure 2:**
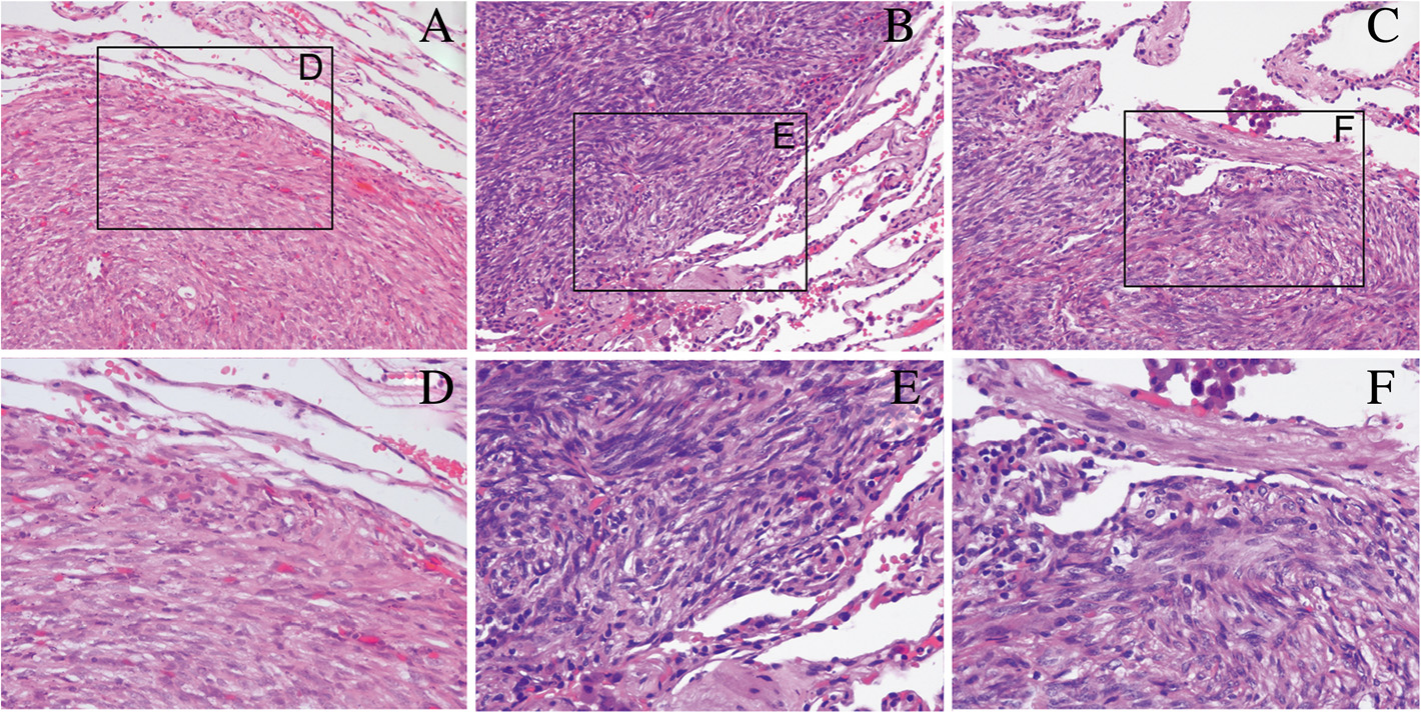
**Histopathologic examination showed tumor consisted of well-differentiated spindle-shaped cells with low nuclear and cellular variance in size and shape; there were no mitotic figures and nuclear atypia.** Case 1 **(A,D)**; Case 2 **(B,E)**; Case 3 **(C,F)** (Magnifications: **A**, **B**, and **C** 20×; **D**, **E**, and **F** 40×).

**Figure 3:**
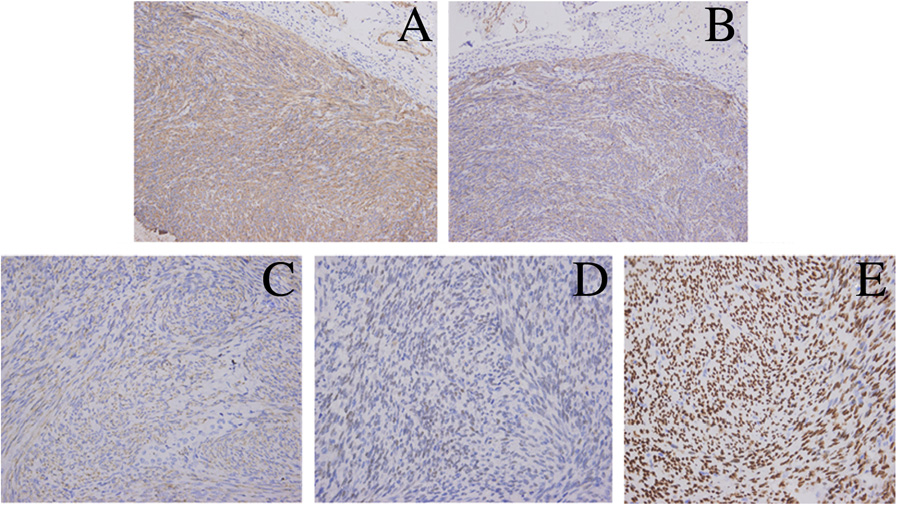
**Immunohistochemical staining of benign metastasizing leiomyoma (BML).** Positive immunohistochemical staining for smooth muscle makers α-smooth muscle actin (**A**, 20×), muscle specific actin (**B**, 20×), desmin (**C**, 40×). Immunohistochemical analysis showed strong expression of estrogen and progesterone receptors in tumor cells (**D** and **E**, 40×).

A 47-year-old woman was referred to our department for multiple nodules in both lungs found by routine examination (Figure 1). She had received subtotal hysterectomy seven years previously for multiple leiomyomas of the uterus and bilateral subtotal thyroidectomy three years previously for benign bilateral thyroid nodules. There was no history of malignancy, 18F-fluorodeoxyglucose positron emission tomography/computed tomography (18F-FDG PET-CT) did not show 18F-FDG uptake in both lung nodules and other parts of the body. To establish a diagnosis, we performed a pulmonary wedge resection by video-assisted thoracoscopic surgery and resected the largest tumor, which was located at the periphery of the left lung. Histopathologic examination and immunohistochemical staining of resected specimens were performed. The pathologic report revealed that tumors were well-circumscribed in resected specimens of the lung, and consisted of well-differentiated spindle-shaped cells with low nuclear and cellular variance in size and shape; there were no mitotic figures and nuclear atypia (Figure 2A, D). Immunohistochemical staining for α-smooth muscle actin (α-SMA), muscle specific actin (MSA), and desmin was positive (Figure 3A–C). The presence of the smooth muscle protein caldesmon and muscle marker calponin were also confirmed by immunohistochemistry in this case. Immunohistochemical analysis also showed strong expression of estrogen and progesterone receptors on the tumor cells (Figure 3D,E). Immunohistochemical analyses are shown in Table 1. The ultimate pathologic results ruled out the possibility of leiomyosarcoma and other metastatic diseases, and confirmed that the pulmonary lesions were BML. The patient was treated with gonadotropin-releasing hormone (GnRH) agonist after thoracic surgery, Zoladex (goserelin acetate) was administered subcutaneously every 28 days for 6 cycles. Nodules in both lungs were hormone sensitive and regressed. After 49 months, CT showed that the lung nodules were unchanged. Data of Case 1 are shown in Table 2.

**Table 1:** Immunohistochemical results

**No.**	**α-SMA**	**MSA**	**Desmin**	**ER**	**PR**	**Calponin**	**Caldesmon**	**Ki67**
1	+	+	+	+	+	+	+	–
2	+	+	+	+	+	+	N/A	3%
3	+	Partial positive	+	+	+	+	+	5%

*α-SMA* α-Smooth muscle actin, *MSA* Muscle specific actin, *ER* Estrogen receptor, *PR* Progesterone receptor.

**Table 2:** Patient characteristics

**No.**	**Age (years)**	**Primary lesion**	**Treatment**	**Interval time (years)**	**Treatment for BML**	**Follow-up (months)**
1	47	Uterine leiomyoma	Subtotal hysterectomy	7	GnRH-analogues	49
2	47	Uterine leiomyoma and ovarian cyst	Hysterectomy + unilateral ovariectomy	17	Unilateral ovariectomy	43
3	53	Uterine leiomyoma	Subtotal hysterectomy	7	Trachelectomy + bilateral ovariectomy	25

### Case 2

**Figure 4:**
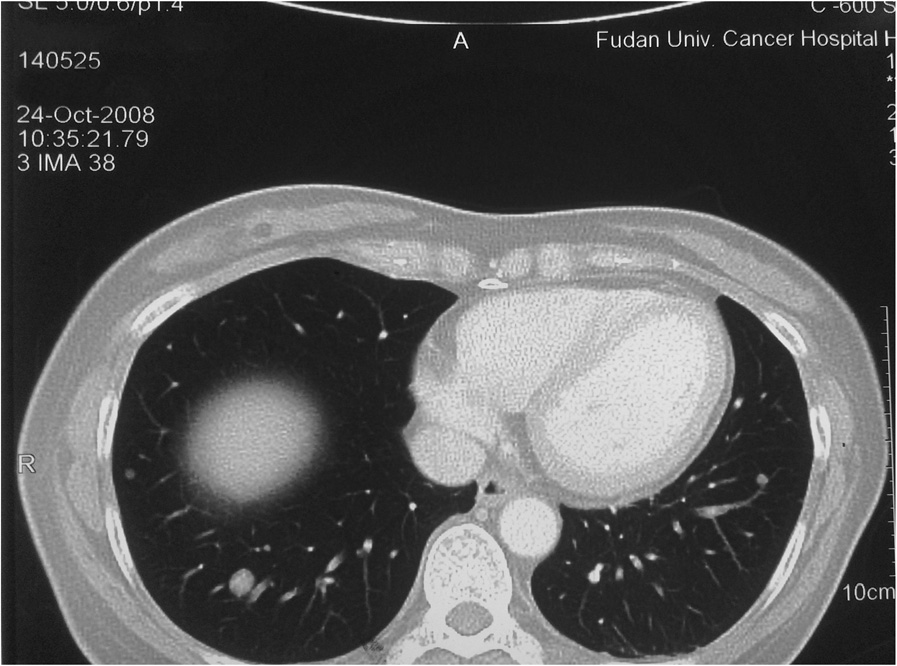
CT of Case 2 shows multiple nodules in both lungs.

A 47-year-old woman had undergone hysterectomy and unilateral ovariectomy for multiple leiomyomas of the uterus and a right ovarian cyst 17 years previously. She complained of abdominal pain and was referred to another unit. A pelvic ultrasound scan showed a left ovary mass, she was prepared for surgery. However, chest CT scan revealed multiple nodules in both lungs during preoperative evaluation (Figure 4). The patient was referred to our department and underwent PET-CT examination, PET-CT did not show any appearance of malignant signs in other parts of the body but moderately intense accumulation of 18F-FDG in parts of the lung nodules. She had no history of malignancy. The patient underwent CT-guided hookwire localization and a pulmonary wedge resection by VATS, pathology showed no cellular atypia or necrosis or no mitotic figures and revealed that lung nodules were BML (Figure 2B, E). Immunohistochemical analyses are shown in Table 1. The patient experienced a unilateral ovariectomy one week after thoracic surgery. Over 43 months follow-up, CT showed that the lung nodules were unchanged. Data of Case 2 are shown in Table 2.

### Case 3

**Figure 5:**
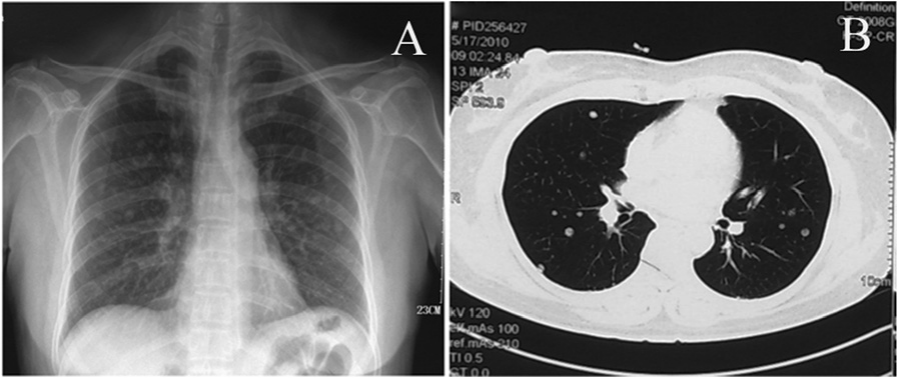
**X-ray and CT images for Case 3.** Posteroanterior chest X-ray reveals bilateral diffuse nodular opacities **(A)**. CT shows multiple nodules in both lungs **(B)**.

Case 3 was a 53-year-old woman with a past medical history of multiple uterine leiomyomas, she had undergone subtotal hysterectomy seven years previously. The patient was asymptomatic and referred to our department for accidental radiologic findings of multiple pulmonary nodules involving both lungs (Figure 5). There was no history of malignancy. PET-CT showed moderately intense accumulation of 18F-FDG in parts of the lung nodules. The patient underwent a pulmonary wedge resection by VATS, pathology revealed that lung nodules were BML (Figure 2C, F). Immunohistochemical analyses are shown in Table 1. She had a trachelectomy and bilateral ovariectomy 11 days later after thoracic surgery. Over 25 months follow-up, the patient appeared well and chest CT scan showed the lung lesions remained stable. Data of Case 3 are shown in Table 2.

## Results

As we have introduced above, the ultimate pathologic results ruled out the possibility of leiomyosarcoma and other metastatic diseases, and confirmed that the pulmonary lesions were BML in all three patients. All patients had a regular follow-up evaluation, including clinical symptom and radiologic evaluation. The lung lesions remained stable in two patients who were treated by surgical castration, and the lung nodules regressed in one patient treated with gonadotropin-releasing hormone analogues. We did not find any additional problems in all patients.

## Discussion

Uterine leiomyoma is the most common type of uterine tumor and is typically considered benign, with a favorable long-term prognosis. However, there are two unusual growth patterns of leiomyoma that are important to recognize. Both BML and disseminated peritoneal leiomyomatosis are found outside the uterus [1]. BML has been described as incidental pulmonary nodules in women with a history of uterine leiomyomas [2]. It occurs predominantly in women of reproductive age [3], and the youngest reported patient was 23 years old [4]. Sometimes, the pulmonary lesions can be found simultaneously with uterine leiomyoma [5]. The mean time period from hysterectomy to the appearance of lung lesions is 15 years [6].

Most BML patients remain asymptomatic, and are revealed to have bilateral diffuse nodular opacities by a routine chest roentgenogram, only a small percentage of patients present with complaints of respiratory disease. The further examination of CT chest scans show multiple varying sized nodular lesions in both lungs; there is no mediastinal lymphadenopathy. Recently, BML was evaluated with metabolic imaging 18F-FDG PET-CT and the absent FDG uptake of the pulmonary lesions ruled out metastasis from extrathoracic malignant tumors or uterine sarcoma [7]. Lin et al. thought that non-avid or mild uptake in 18F-FDG PET-CT without mediastinal and hilar lymph node enlargement or appearance of malignant signs in other parts of the body were criteria in the diagnosis of BML [8]. In the cases presented herein, one patient was PET-negative and two other patients showed moderately intense accumulation of 18F-FDG.

Biopsy through fine-needle aspiration (FNA) may miss the tumor or retrieve only a small number of cells from the lesions, with the risk that the abnormal cells will not be sampled; further, the amount of tissue harvested is usually insufficient. Therefore, VATS was performed instead of FNA biopsy in order to harvest enough tissue for histopathologic and immunohistochemical examination in patients.

Before making the diagnosis of BML, leiomyosarcoma must be excluded. BML consists of well-differentiated spindle-shaped cells with low nuclear and cellular variance in size and shape; there is no disorganized growth pattern and mitotic figures. Besides these indicators, Ki67 index for the BML is less than that for leiomyosarcoma [9,10], and the miRNA, miR-221 is upregulated in leiomyosarcoma. The upregulation of miR-221 expression is an accurate way to differentiate leiomyosarcoma from BML [11]. In the cases herein, the morphological features and immunohistochemical results have provided enough information for the diagnosis of BML; thus, examinations related to leiomyosarcoma and miR-221 were not performed.

Finally, for an accurate diagnosis of BML, a diagnosis of smooth muscle tumors of uncertain malignant potential (STUMP), according to the World Health Organization classification [12], must be ruled out. Uterine smooth muscle tumors that show some worrisome histological features (i.e., necrosis, nuclear atypia, or mitoses), but do not meet all diagnostic criteria for leiomyosarcoma, fall into the category of STUMP [13]. All of our patients were diagnosed with uterine leiomyoma after resection of the uterine tumors and the pathological results of the lung lesions did not show mitotic figures, nuclear atypia, and tumor necrosis. Therefore, we prefer the diagnosis of BML to that of metastasis of STUMP.

BML can have a benign indolent clinical course, with long-term stability [14]. Giove et al. have reported a BML case in a 55-year-old woman still living with lung, skin, lymph nodes, bone, and perhaps brain metastases 14 years after the first uterine myomectomy [15]. Most BML lesions are stable in number, size and clinical symptoms, or progress with low velocity; however, in some cases, the lesions develop a giant tumor mass if no treatment is performed [16]. Some authors have proposed that since BML is hormone-dependent, treatment based on hormonal manipulation through surgical or medical oophorectomy may succeed for BML [17,18]. Despite the presence of positive estrogen and progesterone receptors on smooth muscle cells, there was no significant change in size of BML after 6 to 12 months treatment of tamoxifen, progesterone, and an aromatase inhibitor [2]. Benetti-Pinto et al. have performed the classic treatment of oophorectomy for two patients with BML of the lung, and obtained different outcomes; one achieved an improvement in symptoms, the other did not [19]. Finally, some authors propose that BML might naturally decrease following the menopause [20]. However, cases of pulmonary BML from the uterus in elderly postmenopausal women have been reported [5,21]. Surgical castration combined with hormonal therapy have also been used to treat BML [22]. Thus, the standard treatment for BML is still controversial.

In our limited experiences, further treatment should be offered to these patients following the diagnosis of BML. If possible, surgical excision is the first choice for treatment of BML. However, multiple nodules in both lungs are the most common manifestation in patients with BML and therefore it is impossible to perform the primary excision for these patients; the therapeutic options available are surgical and/or chemical castration. In our patients, both estrogen and progesterone receptors were identified in the lung lesions (Table 2), chemical castration was used in one patient and castration surgery was performed on the other two. The results were satisfactory; the lung lesions remained stable in patients who were treated by surgical castration, especially for the patient who was treated with GnRH analogues, and the lung nodules regressed. Despite performing surgical castration in two patients, we preferred chemical castration rather than surgical castration since it is potentially reversible and can result in complete residual tumor regression [23]. Even though medical castration failed to achieve results, surgical castration may be a good alternative. No further complications were encountered in the clinical follow-up of our patients.

## Conclusions

The diagnosis of BML is based on the medical history of uterine myomas and histopathologic and immunohistochemical examination of lung nodules. Video-assisted thoracoscopic wedge resection is the best way to harvest tissue for diagnosis. The better outcomes in BML seem to call for medical intervention, either chemical or surgical, after diagnosis is made.

## Abbreviations

BML: Benign metastasizing leiomyoma; 18F-FDG PET-CT: 18F-fluorodeoxyglucose positron emission tomography computed tomography; GnRH: Gonadotropin-releasing hormone; MSA: Muscle specific actin; α-SMA: α-smooth muscle actin; STUMP: Smooth muscle tumor of uncertain malignant potential; VATS: Video-assisted thoracoscopic surgery

## Competing interests

The authors have no conflicts of interest or financial ties to disclose.

## Authors’ contributions

HC, LS and YZ conceived and designed the study. HC and YZ carried out the procedures. YC, HH and JZ helped to collect data and draft the manuscript. JZ and SC analysed data and drafted the manuscript. All authors read and approved the final manuscript.
